# The Missed HIV-Positive Children of Ethiopia

**DOI:** 10.1371/journal.pone.0124041

**Published:** 2015-04-16

**Authors:** Elisabetta Pegurri, Elke Konings, Bud Crandall, Hiwot Haile-Selassie, Nelia Matinhure, Warren Naamara, Yibeltal Assefa

**Affiliations:** 1 The Joint United Nations Programme on HIV/AIDS (UNAIDS), Addis Ababa, Ethiopia; 2 Management Sciences for Health (MSH), Addis Ababa, Ethiopia; 3 Consultant, Addis Ababa, Ethiopia; 4 Save the Children, Addis Ababa, Ethiopia; 5 Ethiopian Public Health Institute (EPHI), Addis Ababa, Ethiopia; Brown University, UNITED STATES

## Abstract

**Objective:**

As elsewhere, due to scarcity of data and limited awareness of HIV infection, especially in older children, the HIV epidemic among Ethiopian children appears neglected in national programs (children ART coverage is of only 12% in 2013). This paper estimates the country burden of HIV in older children and investigates the prevalence of HIV in orphans and vulnerable children (OVC) households.

**Design/Methods:**

We analyzed national HIV data for Ethiopia, using Spectrum/ Estimation and Projection Package (EPP) and primary data on children living in households with at least one HIV-positive adult in the Amhara and Tigrai regions. Descriptive analysis of the age and sex distribution of HIV-positive OVC in Ethiopia was performed.

**Results:**

Our Spectrum/EPP analysis estimated the population of HIV-positive children under 15 years old to be 160,000 in 2013. The majority of children (81•6%) were aged five to 14 years. The estimated number of orphans due to AIDS was 800,000. The empirical data from almost 10,000 OVC under 18 years showed 11•9% were HIV-positive, the majority of whom were between five and 10 years old with no significant difference between males and females.

**Conclusions:**

There is a large population of children living with HIV in Ethiopia, the magnitude of which not previously recognized. The majority were vertically infected and never identified nor linked into treatment. OVC represent a reachable group which could account for a substantial proportion of the HIV infected older children. We recommend that HIV programs urgently synergize with social protection sectors and address these children with HIV testing and related services.

## Introduction

Improved coverage and effectiveness of prevention of mother-to-child transmission (PMTCT) programs has reduced new HIV infections among children under 15 years old by 40 per cent globally between 2009 and 2013 [1]. However, there were about 3·2 million children under 15 years of age worldwide living with HIV in 2013, comprising 9·1% of all people living with HIV, with the vast majority residing in Sub-Saharan Africa [1].

Significant challenges remain to reach these children with treatment and care services [2]. In 2013, while 38% of adults living with HIV worldwide received antiretroviral therapy, only 24% of children living with HIV obtained HIV treatment [1].

Recently, an HIV epidemic among older children—presenting for the first time with previously undiagnosed HIV in childhood—has become apparent in many parts of Southern Africa; these are the survivors of MTCT [3, 4]. The limited data on survival among slow progressors among vertically infected children has resulted in failure to anticipate the magnitude of the epidemic [4, 5].

Global estimates indicate there are 17·8 million children who have lost at least one parent to HIV, 85% of whom live in Sub Saharan Africa [5]. We posit that orphans represent a sizeable group that can be reached and may account for a substantial proportion of the population of older HIV infected children.

Orphans are at much increased risk for being HIV-infected compared to other children: a meta-analysis of studies on HIV infection in orphaned populations aged 24 years and younger, which mostly include samples from Sub-Saharan Africa, revealed nearly two-fold greater odds of HIV infection among orphaned youth [6]. Similarly, findings from a population-based HIV counselling and testing initiative in Western Kenya, confirmed high risk of HIV prevalence among orphaned adolescents even in a low prevalence area: orphans were 4·3 (2·2–8·1) times more likely to have HIV while double orphans were 21·4 (10·5–43·9) times more likely to have HIV [7]. However, these studies did not delve into the potential contribution of perinatal transmission to the higher observed HIV prevalence.

In a study by Ferrand et al. [8] on about 500 adolescents attending acute primary care (APC) in Harare, Zimbabwe, maternal transmission was considered to be likely by 80% of the HIV-positive APC attendees; age and sex did not differ by HIV status, but HIV-infected APC attendees were significantly more likely to be maternal or double orphans than their HIV-negative counterparts. In another study on the same dataset, HIV prevalence was reported to be 17%, and infection was independently associated with client-reported orphan hood [9].

Estimates from Sub-saharan Africa show that even in the absence of ART and cotrimoxazole prophylaxis (CTX), 25% of children infected perinatally would still be alive at 10·6 years of age and at 16·9 years for those infected through breastfeeding [10]. In mature epidemics one third of infants are estimated to be slow-progressors with median survival of about 16 years [3].

However, the magnitude of the population of older HIV-positive children, infected through vertical transmission, is insufficiently recognized. In Ethiopia, messages around PMTCT and achieving an “HIV-free generation” through elimination of MTCT has unintentionally led to the false notion that HIV-exposed babies who do not receive treatment will not survive (*Authors observations from HIV stakeholders discourse*, *Ethiopia*). In Ethiopia as elsewhere [11], HIV messages tend to stress sexual transmission of HIV in adults and adolescents and MTCT in relation to infants only. Since there is a high risk of early mortality and this is visible very early for untreated infants, the perception is common that untreated infants will not survive. In a study of attitudes towards HIV counseling and testing (HCT) in the suburbs of Harare, Zimbabwe, HIV-negative participants were not aware that long-term survival following MTCT could occur while adolescents reported that HIV diagnosed at their age must have been sexually acquired [11]. Due to the scarcity of data, limited awareness of possible HIV infection in older children that are not sexually active, combined with denial/stigma and lack of specific training of health care workers, these older children living with vertically acquired HIV appear all but forgotten in national programs.

In Ethiopia, the Government has recently began implementing an accelerated plan to eliminate MTCT [12], including the adoption of lifelong treatment for all pregnant women living with HIV, in response to its high MTCT rates over previous years. The MTCT rate after breastfeeding was estimated at 24% at the end of 2012 among all estimated HIV positive pregnant women in Ethiopia, while it was 30% or higher in earlier years (Spectrum/EPP estimates). Moreover, the proportion of children receiving antiretroviral (ARV) drugs for PMTCT has been negligible [13]. The previous low PMTCT coverage has resulted in a large number of children being born with HIV, with a likely large majority lost to follow-up and no specific mechanisms to trace them to offer them the HIV services they need.

While the evidence is scant, data from HCT services in Tigrai, one of the regions of Ethiopia, from 2008 to 2010 showed high HIV prevalence among the zero to 14 years old group, particularly in the urban areas (9·6% prevalence in 2010 in urban areas compared with 0·7% in rural areas) [14]. Although the number of children tested was relatively small and there are strong selection biases, the HIV prevalence in a population of youth not yet regarded as sexually active—only 1·2% of males and 10·9% of females aged 15–24 years had sexual intercourse before age 15 in Tigrai [15]—is likely to reflect the cumulative result of a PMTCT program lacking in coverage and quality over the years [14].

This paper analyses the country burden of HIV in older children and investigates the prevalence of HIV in OVC households. The paper provides reasons why targeting OVC for HIV testing may offer a sound and feasible strategy to close the gap of undiagnosed HIV in older children and provide children living with HIV with HIV services. OVC is taken as a broader programmatic category including orphans that could not be otherwise singled out.

## Methods

The national burden of HIV infection in children and the number of HIV orphans in Ethiopia was estimated through secondary analysis of national HIV data, using Spectrum/ Estimation and Projection Package (EPP) (version 4·47), a software used globally to produce HIV estimates. The Spectrum/EPP model generates an epidemic curve based on data from antenatal sentinel surveillance and national surveys with seroprevalence testing. In the case of Ethiopia, the data were derived from the national Demographic Health Surveys of 2005 and 2011 and antenatal HIV sentinel surveillance. We used other programmatic data as inputs to model age disaggregated estimates of prevalence and population sizes and the number of orphans due to AIDS among others (see Stover et al. [16] for full details of methods).

We complemented the national estimates with primary data on the number, age, sex and HIV status of children living in households with at least one HIV-positive adult supported by the National Network of Ethiopian Women Ethiopia (NNPWE) in the Amhara and Tigrai regions of northern Ethiopia. NNPWE is a member association of HIV-positive women that provides community-based care and support to HIV-infected and affected families through a network of HIV-positive volunteers. Each volunteer provides home-based support to highly vulnerable HIV infected households in their own community.

The data were collected and reported by 350 NNPWE volunteers in August 2013 through a partnership between NNPWE and the USAID Ethiopia Network for HIV/AIDS Treatment Care and Support (ENHAT-CS) program, which is funded by PEPFAR and implemented by a Management Sciences for Health (MSH) led consortium of international and Ethiopian organizations, including the NNPWE. As the data is from highly vulnerable HIV-infected households, the analysis reflects a select sub-population that would be expected to have higher HIV prevalence than that found in the general population. However, the descriptive analysis of this data provides evidence of the age and sex distribution of HIV-positive OVC in Ethiopia.

## Results

Ethiopia’s HIV prevalence in the adult population was 1.5% in 2011 [15]. No population based HIV prevalence surveys exist for children below the age of 15. Modelled HIV prevalence among zero to 14 year olds was 0·4% in 2013 (Spectrum/EPP estimates). Of the 785,000 people currently living with HIV in Ethiopia (PLHIV), 158,400 (20%) were estimated to be children [17].

**Fig 1:**
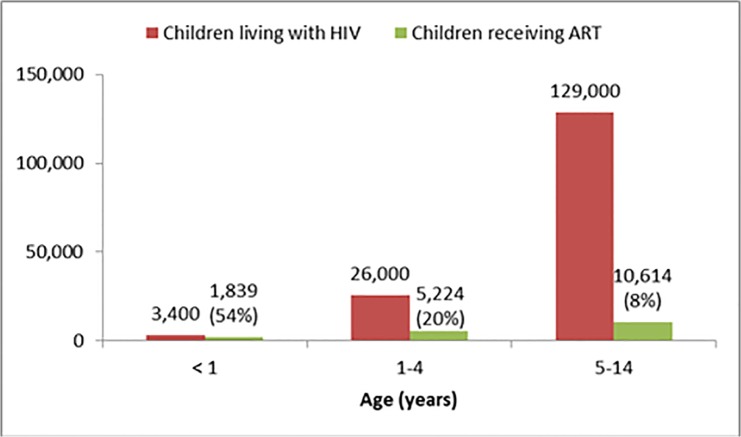
Estimated number of children younger than 15 years living with HIV and receiving ART, by age, Ethiopia, 2012 (A). (A)EPP/Spectrum estimates for children living with HIV; Estimates from the Federal HIV Prevention and Control Office (FHAPCO) and PEPFAR data for children receiving ART.

When modelling the age distribution of PLHIV in Ethiopia, 3,400 (2·1%) children were under one year old; 26,000 (16·4%) children aged one to four years; and 129,000 (81·6%) children aged five to 14 years (Fig 1).

In the same time period, Ethiopia had only 17,677 HIV-positive children enrolled on ART [13] (12% coverage).

Fig 1 portrays the age distribution of children receiving ART.

**Fig 2:**
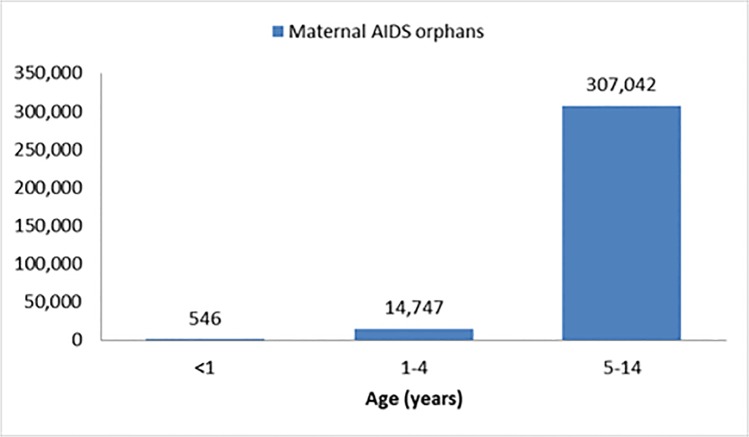
Number of maternal AIDS orphans, by age, 2013 (A). (A) EPP/Spectrum estimates, 2012.

A vital set of complementary data when looking at issues of HIV in children in Ethiopia concerns orphans. In 2013, there were an estimated 800,000 orphans due to AIDS in Ethiopia out of 3,700,000 estimated orphans [17]. Of those, 300,000 were estimated to be double orphans (Fig 2). In a setting with low coverage of PMTCT and AIDS treatment, orphans, in particular maternal orphans, have a higher chance of being HIV infected; therefore, a significant part of the HIV positive children may be found among this group.

**Fig 3:**
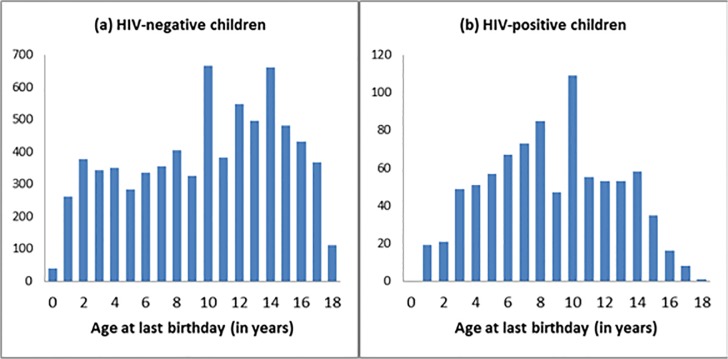
Age distribution of (a) HIV-negative and (b) HIV-positive among OVC supported by NNPWE through the USAID ENHAT-CS program, Amhara and Tigrai regions, Ethiopia, 2013.

Complementing the findings of the Spectrum modeling, we obtained household level data for a total of 11,374 OVC under 18 years of age, from 9,961 households (i.e. an average of 1·1 children per household). Among the 11,374 children, 10,602 (93%) were reported (by the adult care givers in their households and/or NNPWE) knowing their HIV status and of those, 1,124 (11·9%) were HIV-positive. The age distribution was available for 8,724 (82%) of these children and shows that both OVC with HIV and those without HIV were found across all ages. The numbers, particularly among HIV-positive OVC steadily declined after the age of 14years (see Fig 3). In addition, there is a blip at age ten, for both HIV-positive and HIV-negative children, that might have been partly due to age heaping.

**Fig 4:**
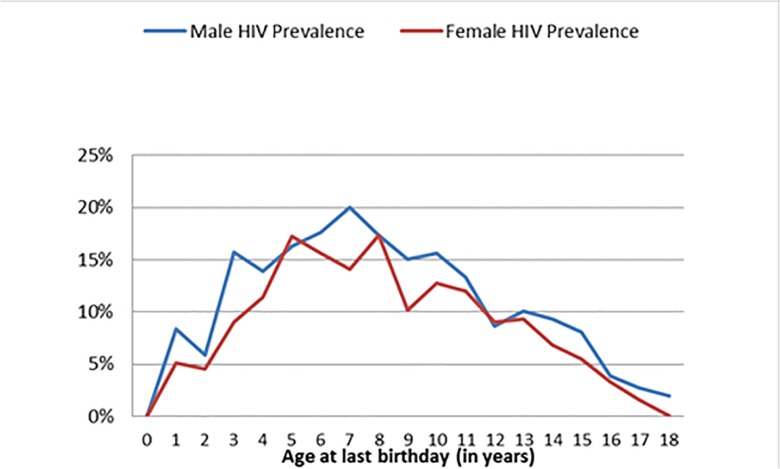
HIV Prevalence by Age and Gender of children living in households with at least one HIV-positive adult (A). (A)Data collected on OVC supported by NNPWE through the USAID ENHAT-CS program, Amhara and Tigrai regions, Ethiopia, 2013.

This age distribution is further borne out by age-specific HIV prevalence which peaked in the five to nine year old age group, for both boys and girls (Fig 4). This is similar to EPP/Spectrum modelled age distribution of HIV positive children where the majority is above five years old.

## Discussion

The magnitude of the HIV burden in older, not yet sexually active children has never been recognized or documented in Ethiopia. Our EPP/Spectrum analysis brought to light the large population of almost 160,000 children between five and 15 years old estimated to be living with HIV in Ethiopia in 2013. This represents 20% of all PLHIV estimated to live in Ethiopia. PMTCT programs started to scale up from about 2006 in Ethiopia. The smaller number of children infected at younger ages (0–4) is likely due to the higher coverage and effectiveness of PMTCT in the last five years. The larger number of infected children at older ages reflects that lack of PMTCT programs when mother to child transmission would have been high. The low level of antenatal care attendance has been, and partly still is, one of the main challenges to reach high PMTCT coverage.

The modeled estimates are supported by the empirical data that we collected from almost 10,000 households with at least one HIV-positive adult in the Amhara and Tigrai regions, showing that 11·9% of children under the age of 18 years in those households were infected with HIV; the majority of the HIV-positive children were between five and ten years old. The overall trend by age appeared similar between boys and girls, suggesting that infection was the result of survival rather than early onset of sexual relations. If the main mode of transmission was sexual we would expect a larger prevalence in the female group and in older ages than their male counterparts due to their earlier sexual debut, as evidenced by the HIV prevalence among 15–24 years of 0·5% for females and 0·1% for males in Ethiopia [15]. The significant drop off in the number of OVC with HIV after the age of 14 years, but not in the number of HIV-negative OVC in the affected households also indicates that the HIV-positive children are survivors of vertical transmission. This finding is consistent with earlier estimates that the median survival of slow progressors is 16 years [3] and indicates the urgency of having OVC tested early.

A limitation of the study is that the data are reported and not verified with biomedical measures (blood testing). However, there is no incentive for families and care givers to lie about the HIV status of their children and since NNPWE is a well-respected and established local NGO caring for families affected and infected with HIV, we believe the information to be truthful.

However, although the estimated number of HIV-infected children under 15 years of age is large—approximately 160,000 in 2013—finding them has proven challenging, in part because so many critical questions remain unanswered in the Ethiopia context including: Who and where are these children? Which—if any- HIV testing services are available for older children and how easy is it for them to access existing services? How to identify and provide adequate support for children that are not living in family structures and so excluded from family centric approaches and are not otherwise accessing health services?

Furthermore, in Ethiopia there are legal barriers to access HCT for children below 15 years of age. Current guidelines for Ethiopia state that HCT for children under 15 years shall only be done with the knowledge and consent of parents or guardians, with the exception of children aged 13–15, who are in specific circumstances: married, pregnant, commercial sex workers, street children, heads of families, or sexually active [18]. The latter are regarded as “mature minors” who can consent to HIV testing. It appears from the guidelines that the focus is on sexually active children, overlooking those who were vertically infected and still alive.

As with any medical intervention, HIV testing is an ethical priority if it is clinically necessary to support life-saving treatment, such as initiation of ART. In all circumstances the best interests of the child should be the guiding principle. Policy on the testing of children should address the specific circumstances of OVC [19]. Lack of clarity about age of consent and procedures for HCT may inhibit providers from offering services and limit access [20]. Policy and guidelines may need to be revised to reflect who is responsible for testing and referral, to clarify age-appropriate consent and disclosure procedures, and to account for the special circumstances of children without parents or guardians [19]. National modeling data showed that only 12% of HIV-positive children 15 years and younger are on ART. The widest gap in term of ART coverage is among children in the older age groups. Delaying HIV testing can have serious implications for the health of children living with HIV. Access to ART improves immunological response and reduces opportunistic infections and co-morbidities [21]. While recognizing the complexities, it is important to facilitate greater uptake of HIV testing amongst children and in particular those groups of children that might have a higher prevalence of HIV. In Ethiopia, reaching orphans (within the larger beneficiary group of OVC)—of whom about 20% are estimated to be orphans due to AIDS [17]—with HCT services may provide the greatest returns in identifying and responding to the HIV needs of children.

To improve case finding and provision of HIV services to the missing HIV-positive children of Ethiopia, the pediatric ART delivery model needs to be re-thought. The family-based model is essential but not sufficient and there is a need for improved integration with the social protection sector and cooperation with actors serving the broader arrays of vulnerable children needs.

To address the diverse and complex needs of orphans and other vulnerable children in Ethiopia, current interventions cover food/nutrition, shelter and care, legal protection, health care, psychosocial support, education and economic strengthening. Health care services include provision of primary care, monitoring health, immunization and HIV prevention. While treatment for HIV positive children is captured, there is no pro-active approach to providing HIV testing to children who are receiving other OVC services [22]. In addition to identifying children living with HIV, the HIV testing program would further provide opportunities for increased HIV awareness.

Although only a small fraction of OVC receive support services in Ethiopia [13] they still represent a sizable population of children—more than 500,000 in 2013 according to PEPFAR programmatic data. These children that are already enrolled in a program represent a good entry point for actual promotion of HIV testing and provision of treatment. In Zimbabwe, an algorithm was developed and evaluated to be used by health workers to identify children likely to be HIV positive and one of the significant predictive factors is orphanhood, a finding that highlights the feasibility of selectively testing [9]. Although we acknowledge that OVC in Ethiopia are a much broader category than orphans and includes other vulnerable children [22], it still represents a programmatically feasible way to identify HIV positive children over universal testing outside the health facilities (beyond provider-initiated HIV testing and counseling and tracing of children of HIV positive individuals) and already exists as a beneficiary category for programs. It would be questionable to single out orphans within the broader category of OVC.

Harmonization of pediatric ART treatment with adult regimens and optimization (simpler, easier to administer such as fixed-dose combinations, and affordable) of available formulations may facilitate the actual implementation of ART programs once children are identified [23]. As with other chronic conditions it will be critical to look at issues of long term treatment; monitoring and management of the response to ART [24]. Compared with adults, adolescents in southern Africa are less adherent to ART [25] and the percentage of non-adherence is higher among children who have lost one or both parents compared to children with both parents alive [26].

Orphans and vulnerable children may face additional challenges, such as multiple caretakers, or lack of them, the fact that they may be themselves caretakers, stigmatization, depression, fear of disclosure, poverty, and inadequate nutrition [24]. Care provision for children living with HIV should encompass the clinical, adherence, mental health, sexual health and social spheres [4]; the latter of particular significance for OVC.

Moreover, as soon as children transition into adolescence and adulthood, additional risks of sexual transmission of HIV needs to be taken into account. In the case of Ethiopia, risks of sexual transmission might be even higher given that most of these children are unaware of their HIV status.

## Conclusion

This paper demonstrates that there is an estimated large number of HIV positive children in Ethiopia, mainly in the older age groups that are neither identified nor served. A substantial proportion of these children may be found among orphans—and more broadly (taking programmatic issues into account) OVC.

If there is an urgent need to put into place robust systems for better follow-up of mother-baby pairs when mothers are HIV positive and identifying and testing the children of all HIV-positive adults attending HIV services, there is also need to go beyond the health sector and provision of HIV testing in health facilities. A more comprehensive strategy is required to identify HIV-positive children and address their needs. Synergies with social protection and child protection stakeholders and programs need to be sought to integrate HIV testing and provision of HIV treatment into broader programs of care and support.

Although more studies among OVC to assess HIV prevalence and access to services are warranted, it is urgent and vital to advocate that HIV services for OVC in Ethiopia should expand beyond HIV prevention programs such as IEC/BCC and include proactive case finding and access to ART for those in need. An important number of current OVC have been vertically infected but remain asymptomatic; they critically deserve the chance to receive HIV testing and referral to ART if needed. HIV diagnosis, treatment, care and support should be integrated in the package of economic and psychosocial support that is particularly important for the estimated 800,000 children who have lost one or both parents to AIDS in Ethiopia. Only an integrated approach can facilitate access and likely support long-term adherence to medication and ensure the continuity of care for those children.

### Disclaimer

The contents are the responsibility of the authors and do not necessarily reflect the views of USAID or the United States Government nor the Joint United Nations Programme on HIV/AIDS (UNAIDS).

## Supporting Information

S1 Data Empirical data on OVC supported by NNPWE through the USAID ENHAT-CS program, Amhara and Tigrai regions, Ethiopia, 2013. (XLSX)
